# Process evaluation of a multicomponent dyadic intervention study with exercise and support for people with dementia and their family caregivers

**DOI:** 10.1186/1745-6215-15-401

**Published:** 2014-10-22

**Authors:** Anna-Eva Prick, Jacomine de Lange, Netta van ‘t Leven, Anne Margriet Pot

**Affiliations:** Department of Clinical psychology and the EMGO institute for Health and Care Research, Faculty of Psychology and Education, VU University, Van der Boechorststraat 1, 1081, BT Amsterdam, The Netherlands; Research Centre Innovations in Care, Rotterdam University of Applied Sciences, Rochussenstraat 198, 3015, EK Rotterdam, The Netherlands; Program on Ageing, Institute on Mental Health and Addiction, Da Costakade 45, 3500, AS Utrecht, The Netherlands

**Keywords:** Dementia, Intervention, Process evaluation, Caregivers, Exercise, Support, Mood

## Abstract

**Background:**

A randomized controlled trial of a multicomponent dyadic intervention (a translated and adapted version of an intervention that has been shown to be effective for people with dementia in the USA) was performed. The exercise and support intervention was intended to reduce depressive symptoms of people with dementia and their caregivers. The purpose of this process evaluation is to create in-depth insight into the delivery of the intervention and the effect analysis, to prevent drawing inappropriate conclusions on the efficacy or effectiveness of the intervention, and to formulate recommendations for future studies on complex geriatric interventions.

**Methods:**

Qualitative and quantitative data were collected. The process evaluation was performed according to the model presented by Reelick and colleagues, which encompasses the following three process components: (1) success rate of recruitment and quality of the study population; (2) the quality of execution of the complex intervention; and (3) the process of acquisition of the data.

**Results:**

The study design met high research standards and the intervention was carefully delivered. Evaluation of the study population quality revealed a profound recruitment process resulting in a reasonable sample size. Attrition rate during follow-up was acceptable. With regard to the evaluation of the intervention quality, most interviewed participants experienced benefits of the intervention. Attendance at the home visits was high and attrition to homework was moderate. Evaluation of the data acquisition showed the positive value of the use of a mixed design; qualitative analysis of the intervention revealed outcomes not measured in the quantitative analysis.

**Conclusions:**

The process evaluation revealed a carefully and soundly performed study. The mixed design contributed to valuable insights. However, there were some restrictions worth considering. The intervention components may have a different feasibility by moderate attrition to homework and some negative experiences of participants, which may be an indication of too intensive an intervention for this frail population in this specific country. As a result, the results of the statistical effect analysis should be interpreted with caution.

**Trial registration:**

The study has been registered at the Netherlands National Trial Register: NTR1802, registration date 6 May 2009.

## Background

The negative impact of dementia has been widely studied: psychological, behavioral, and physical symptoms decrease the quality of life of both people with dementia and their family caregivers [1, 2]. Since most people with dementia live at home with the help of a family caregiver for as long as possible, community-based interventions are much needed. As an alternative to pharmacological treatments with limited effectiveness and possible adverse effects [3], systematic reviews have demonstrated the effectiveness of nonpharmacological or psychosocial treatments for behavioral and psychological symptoms in people with dementia [4–7]. Psychosocial interventions that address both the person with dementia and their caregiver (so-called dyadic or combined interventions) have the potential to reduce the frequency and severity of behavioral and psychological symptoms of dementia as well as negative caregiver reactions [7–12], especially when delivered individually at home using multiple components (multicomponent interventions).

Considering the potential of multicomponent dyadic interventions, we performed an exercise and support intervention study for people with dementia living at home and their caregivers that was primarily intended to reduce depressive symptoms. To investigate the effects of the multicomponent dyadic intervention study, a randomized controlled trial (RCT) was executed [13]. This intervention was largely based on that of Teri and colleagues [14], which showed beneficial effects for people with dementia in the USA. Time and money might be saved when an intervention program that has already been developed, piloted and evaluated on effectiveness in one country can be directly proven to be effective in the healthcare context of another country.

Complex interventions include several components and are thus subject to more variation making replication more difficult, in particular for this vulnerable target population with dementia. To interpret any outcomes, evaluating the feasibility of a complex intervention study is essential before execution of any quantitative outcome effect analyses. Such a process evaluation is needed for drawing the appropriate conclusions on the effectiveness of the intervention study. Therefore, process evaluations should be performed to the same high methodological and reporting standards as the clinical trial and its outcomes [15]. However, many complex interventions are not evaluated to a standard and, when a process evaluation is present, the evaluation components differ from study to study. Possible causes are a lack of standardized measurement instruments for process evaluations and the fact that these evaluations may be time consuming and regarded to be of less interest than the effect analyses [15]. In dementia studies especially, the burden on dementia people and their caregivers because of additional measurements may hamper a process evaluation. However, for complex interventions in this vulnerable target population with dementia, accurate insight into the process is very important and has to be accurately planned [15].

There are different guides, frameworks and models available for performing a process evaluation [15–18]. Although these models provide an extensive evaluation of the intervention, they do not include an evaluation of the study [16, 18] or they do not describe in detail how to carry out a process evaluation [17]. Both components are important in understanding the results of an effectiveness study [19]. The model presented by Reelick and colleagues [15] integrates both an evaluation of the intervention and the study, and is specifically designed for evaluating complex geriatric interventions, including a detailed process evaluation. The primary goal of the model presented by Reelick and colleagues is to create in-depth insight into the performed intervention and effect analysis, to prevent drawing inappropriate conclusions on the efficacy or effectiveness. Furthermore, it gives information on barriers and facilitators, and experiences from participants of this intervention study, resulting in recommendations for future studies or replications of complex geriatric interventions.

In this article, a process evaluation of this RCT intervention study is carried out according to the three questions formulated by Reelick and colleagues [15]: 1) what is the quality of the success rate of recruitment and the quality of the study population; 2) what is the quality of execution of the intervention; and 3) what is the quality of the process of acquisition of the data?

## Methods

### Randomized controlled trial: population, randomization and blinding

For the RCT study both people with dementia living in the community and their family caregivers were included. Inclusion criteria for people with dementia were a diagnosis of dementia made by a physician (for instance, a general practitioner, psychiatrist, geriatrician or neurologist), a minimum age of 55 years, and living at home with a caregiver willing to participate in the home visits. For people with dementia who were interested in the study there was no requirement for permission from their physician to participate in this study. Exclusion criteria for people with dementia were use of antidepressants, presence of psychotic symptoms, mini-mental state examination (MMSE) score <14 and receiving more than 2 days of respite care in a day-care facility at the start of the intervention. Exclusion criteria for caregivers were physical disorders that hampered assistance with the exercises, presence of psychotic symptoms and use of antidepressants. Furthermore, caregivers needed to have at least some depressive symptoms (Centre for Epidemiologic Studies-Depression score >5) and enough understanding of the Dutch language to be included.

After initial assessment, a total of 111 people with dementia and their caregivers (dyads) living in the community were randomly allocated to the intervention group (n = 57) or to the comparison group receiving minimal intervention (n = 54). An independent researcher made the random allocation schedule (in blocks of 20 dyads), using Random Allocation Software, version 1.0 (M. **Saghaei**, Isfahan University of Medical Sciences, Isfahan, Iran) [20]. Self evidently, dyads and their intervention instruction coaches were aware of the treatment assigned. Although examiners were blinded to the group allocation and dyads were asked not to disclose their group allocation at the start of each measurement, group allocation became clear to examiners in practice.

Outcomes were measured three times: before intervention, after 3 months (post-intervention), and after 6 months (follow-up). For the intervention group only, there was a second follow-up measurement at 12 months. Primary outcome variables were physical health (people with dementia) and mood (people with dementia and caregivers). Secondary outcome measurements were directed at burden (caregivers) and behavior problems and cognition (people with dementia).

The study design has been extensively described elsewhere [13].

### Intervention

The intervention was largely based on an intervention of Teri and colleagues [14], which showed beneficial effects for people with dementia. That intervention combined a physical exercise program (30 minutes of daily moderate-intensive exercise) for people with dementia and teaching caregivers how to manage behavioral problems (Activating events Beliefs Consequences (ABC) training) and to identify pleasant activities.

After a pilot study, using a translated version of the Teri intervention, we made changes to the intervention components and duration to adapt the intervention to the Dutch care situation. Because the evaluation of the pilot study showed that a proper execution of the intervention took more than 1 hour, we decided to concentrate on physical exercise, education and pleasant activities training and to drop the time-consuming ABC training. This choice was further motivated by the fact that cognitive reframing (based on the ABC theory) has already been shown to be effective [21], whereas the effectiveness of physical exercise and pleasant activities training for people with dementia and their caregivers in the community is less well studied. In addition, we used elements of a Dutch exercise program for people with dementia and their caregivers designed by Dutch physiotherapists [22]. To improve the attractiveness of the exercises for people with dementia, we used additional materials like a ball, weights and elastic bands, which we integrated with the original exercises. Furthermore, in contrast to the intervention of Teri, we taught caregivers not only to plan pleasant activities for people with dementia but also for caregivers themselves to reduce psychological distress. To the user manual, we added a list of pleasant activities ideas especially for caregivers to stimulate planning pleasant activities for themselves. The number of home visits was decreased to eight instead of the original 12 (in order to comply with Dutch health insurance regulations about the number of home visits typically reimbursed), and the frequency of home visits in the first month was decreased to one instead of two home visits per week. In the Netherlands, for people with dementia living in the community and interested in this intervention it is common to receive respite care in a day-care facility for 1 or more days. In our sample, most people with dementia received 2 days respite care in a day-care facility, so more than one session per week would have been too time consuming in the daily life of the dyads.

In the final adapted intervention, dyads allocated to the experimental group received an intervention consisting of two components: an exercise component and a support component (Table 1). Each aspect of the protocol was implemented with both the person with dementia and the caregiver present. The goal of the exercise component was to motivate dyads to complete 30 minutes of active exercise at least 3 days a week. In line with the intervention of Teri and colleagues [14], four types of exercises were instructed and practiced: flexibility, strengthening, balance and endurance exercises. The exercises were introduced gradually, session-by-session by an individual coach. Alongside the instruction visits, all dyads received a user manual with pictures of the exercises and easy-to-read instructions. This manual also included session-specific worksheets with information and psycho-education for each visit. The support component, directed at both the person with dementia and the caregiver, included three elements: (a) psycho-education; (b) communication skills training; and (c) pleasant activities training. The goal of psycho-education was to educate the dyad about dementia (that is, its impact on the person with dementia and caregiver and how to deal with it). The communication skills training taught techniques for facilitating the dyad’s communication such as tips about tone of voice and speed. Pleasant activities training stimulated the inclusion of pleasant activities in daily life for both the person with dementia and the caregiver. Dyads were asked weekly to plan pleasant activities for both partners. A personal coach visited the dyads in their own homes for eight 1-hour home visits over 3 months. In the first month the dyads were visited weekly, followed by bi-weekly home visits over the next 8 weeks. The coaches were five MSc students of the Department of Clinical Psychology, VU University, who followed a special training program on geropsychology and extensive training to provide the present intervention. To ensure that all coaches followed the treatment protocol in the same way, all coaches were supervised by a psychologist during three visits, and three intermediate meetings were planned with all coaches under supervision of the psychologist.

**Table 1:** **Summary description of total intervention: eight home visits including homework**

Week 1	
	*Home visit 1*
	Acquaintance and explanation of the different components of the intervention by the coach. Introduction of first flexibility exercises
	**•** Physical exercise homework: complete 30 minutes of flexibility exercises on at least 3 days by both person with dementia and caregiver
Week 2	
	*Home visit 2*
	Training communication skills. Psycho-education about specific behavior disturbances in dementia, such as depression, delusions, anxiety and agitation. Demonstration and practice of flexibility exercises.
	• Physical exercise homework: complete 30 minutes of flexibility exercises on at least 3 days by both person with dementia and caregiver
Week 3	
	*Home visit 3*
	Introduction of pleasant activities training. Introduction and practice of first strength exercises.
	• Physical exercise homework: complete 30 minutes of flexibility and strengthening exercises on at least 3 days by both person with dementia and caregiver
	• Pleasant activities homework: plan 2 to 3 pleasant activities for person with dementia and monitor mood
Weeks 4 and 5	
	*Home visit 4*
	Planning 2–3 pleasant activities for person with dementia in the next 2 weeks. Demonstration and practice of flexibility and strength exercises.
	• Physical exercise homework: complete 30 minutes of flexibility and strengthening exercises on at least 3 days a week by both person with dementia and caregiver
	• Pleasant activities homework: plan 3 pleasant activities for person with dementia and monitor mood
Weeks 6 and 7	
	*Home visit 5*
	Psycho-education about coping strategies for caregiver. Introduction of balance exercises.
	• Physical exercise homework: complete 30 minutes of flexibility, strengthening and balance exercises on at least 3 days a week by both person with dementia and caregiver
	• Pleasant activities homework: plan 3 pleasant activities for person with dementia and monitor mood
Weeks 8 and 9	
	*Home visit 6*
	Psycho-education about the importance of respite care for caregiver. Planning pleasant activities for caregiver. Introduction of endurance exercises.
	• Physical exercise homework: complete 30 minutes of flexibility, strengthening, balance and endurance exercises at least 3 days a week by both person with dementia and caregiver
	• Pleasant activities homework: plan 3 pleasant activities for caregiver and monitor mood
Weeks 10 and 11	
	*Home visit 7*
	Psycho-education about dealing with stress reaction for caregiver. Teaching caregivers effective instructions to encourage exercises and to avoid problems.
	• Physical exercise homework: repeat and continue learned flexibility, strengthening, balance and endurance exercises for 30 minutes on at least 3 days a week by both person with dementia and caregiver
	• Pleasant activities homework: plan 3 pleasant activities for both partners and monitor mood
Week 12	
	*Home visit 8*
	Special attention is paid to maintain exercise training and pleasant activities training in future.
	• Physical exercise homework: repeat and continue learned flexibility, strengthening, balance and endurance exercises for 30 minutes on at least 3 days a week by both person with dementia and caregiver
	• Pleasant activities homework: plan 3 pleasant activities for both partners and monitor mood

### Comparison group with minimal intervention

Besides the usual care, dyads allocated to the comparison group received a minimal intervention. This minimal intervention consisted of information bulletins with general information such as car driving and technology support. Every month, these dyads received one bulletin (three in total) and a phone call by one the coaches (three in total). The goal of these 10-minute phone calls were to listen to the caregiver and show empathy.

### Process evaluation design

We structured and evaluated our process evaluation of the RCT study and the intervention according to the framework presented by Reelick and colleagues [15]. Reelick structured the process evaluation for trials on complex interventions into three components (used as a basis for our three defined research questions): (1) success rate of recruitment and quality of the study population; (2) the quality of execution of the complex intervention; and (3) the process of acquisition of the data (Table 2). To assess each process component we made use of a mixed-method design, in which we analyzed quantitative and qualitative data. Quantitative data were used to answer research questions 1 and 3 on the quality of study population and data acquisition. Qualitative data were mainly used for answering research question 2 on the quality of the intervention components. Table 3 shows the variables operationalizing the three process components of our multicomponent intervention study.

**Table 2:** **Process evaluation components and related process measures of a complex intervention according to Reelick and colleagues** [15]

Process components	Process measures
Study population	1. Recruitment and selection rate
	2. Barriers and facilitators in recruitment and selection process
	3. Follow-up: attrition rate
	4. Barriers and facilitators for follow-up
Multiple components	1. Quality of delivery of the interventional components
	2. Barriers and facilitators for delivery of interventional components
	3. Adherence to interventional components
	4. Barriers and facilitators for adherence to interventional components
	5. Experience of participants and instructors with interventional components
Data acquisition	1. Outcome measures: coverage of interventional components
	2. Completeness of data collection
	3. Barriers and facilitators for data collection

**Table 3:** **Process variables collected for the process evaluation of a multicomponent dyadic intervention study according to Reelick and colleagues** [15]

Process measures	Process variables
**Study population**	
1. Recruitment and selection rate	a) Number of eligible persons in screened population
	b) Number of dyads from the sample of eligible persons
	c) Number of dyads versus aimed number
2. Barriers and facilitators in recruitment and selection process	a) Difference in baseline characteristics between nonparticipating and participating eligible dyads
	b) Motivation of nonparticipating and participating eligible dyads
	c) Experience with recruitment and selection
3. Follow-up: attrition rate	Number of dyads completing follow-up versus number started
4. Barriers and facilitators for follow-up	Reasons for drop-out and motivation for continued participation
**Multiple components**	
1. Quality of delivery of the interventional components	a) The part of each component and the home visits delivered by the coaches
	b) Satisfaction with delivery of home visits
2. Barriers and facilitators for delivery of interventional components	Reasons for diverging from or applying intervention components
3. Adherence to interventional components	a) Number of home visits followed
	b) Intervention components (partly) followed
	c) Homework adherence
4. Barriers and facilitators for adherence to interventional components	Motivation for (lack of) attendance and compliance
5. Experience of participants and instructors with interventional components	a) Perceived benefit
	b) Strong and weak aspects of the interventional components and total intervention
**Data acquisition**	
1. Outcome measures: coverage of interventional components	Average number of outcomes per component
2. Completeness of data collection	a) Number and characteristics of missing data
	b) Feasibility of outcome measures
	c) Reasons why data were missing
3. Barriers and facilitators for data collection	Comparison of qualitative and quantitative effectiveness data

#### Quantitative data

Quantitative data were collected using the research database. As shown in Table 3, to evaluate the study population the success rate of recruitment, selection rate and attrition rate were defined by a description of the number of eligible persons in the screened population, the number of participants from the sample of eligible persons, the number of participants versus the aimed number, differences in baseline characteristics between nonparticipating and participating eligible persons and the number of dyads completing follow-up versus the number who started. To evaluate the data, outcome measures and completeness of data collection were defined by a description of average number of outcomes per component and number and characteristics of missing data.

### Qualitative data

We used data from semi-structured interviews, conducted by the first and third author, with eleven dyads who completed the eight home visits of the intervention, and interviewer reflection notes made directly after the interviews. In addition, daily exercise and pleasant activities logs of all caregivers (n = 57) in the intervention group were used. The eleven dyads were selected using the purposeful sampling technique: we invited dyads with diverse adherence to exercise and pleasant activities planning, and gender, age, dementia type and type of relationship between caregiver and person with dementia, as shown in Table 4. No dyads refused to participate in the interviews. Interviews continued until saturation was reached. The semi-structured interview was guided by a series of pre-determined questions to explore participants’ experiences of the intervention components (exercise and support), with flexibility in the order in which they were asked to allow the interview to flow. Questions included: Could you describe your experiences with regard to the physical exercises? Could you describe your experiences with pleasant activities planning? Could you describe your experiences with regard to the communication skills training?

**Table 4:** **Characteristics of eleven interviewed dyads for qualitative study**

Dyad					Person with dementia				Caregiver		
Dyad number	Relation	Individually interviewed	Adherence to exercise homework	Adherence to pleasant activities homework	Gender	Age	Education	Dementia type	Gender	Age	Education
**1**	Couple	CG = yes/PD = no	Low	High	Female	85	Moderate	Vascular dementia	Male	85	High
**2**	Couple	No (on request)	High	High	Female	70	High	Frontotemporal	Male	75	Moderate
**3**	Couple	Yes	High	Moderate	Male	62	High	AD	Female	61	High
**4**	Couple	Yes	High	Moderate	Male	81	High	AD	Female	74	High
**5**	Couple	No (on request)	High	High	Male	67	Moderate	AD	Female	69	High
**6**	Couple	No (on request)	Low	High	Female	85	Low	AD	Male	85	High
**7**	Couple	Yes	Moderate	Moderate	Male	81	Moderate	AD	Female	76	Low
**8**	Couple	No (on request)	High	Low	Male	67	High	AD	Female	68	High
**9**	Mother/daughter	CG = yes/PD = no	Moderate	Moderate	Female	83	Low	Vascular dementia	Female	53	High
**10**	Couple	No (on request)	Moderate	Moderate	Female	73	High	AD	Male	76	High
**11**	Couple	No (on request)	High	High	Male	70	High	Vascular dementia	Female	69	High

AD, Alzheimer's disease; CG, caregiver; PD, person with dementia.

The participants were interviewed at home. When possible, people with dementia and caregivers were interviewed separately without each other’s presence. Because most dyads lived together, the non-participant was asked to stay in another room. Some dyads wanted to be interviewed together (“We have no secrets from each other”, CG 2). Field notes were made directly after the interview on a semi-structured form. Digital recordings were directly reviewed upon the return of the interviewers. There were no recording failures. Regular meetings were held between first, second and third author to discuss important themes and any conceptual issues. No repeat interviews were carried out to avoid excessive burden on participants.

### Analysis

The interviews were audio taped and transcribed verbatim. The transcripts were initially coded using open codes based on the words the participants used. Coding started after the first interview so that any emergent themes could be incorporated into subsequent interviews. To avoid bias, the first three authors independently coded the data. The coding process was supported by ATLAS.Ti (version 6.2; ATLAS.ti GmbH, Berlin, Germany). Differences were discussed until consensus was reached. Interviewer reflection notes and logbooks of all dyads randomized to the intervention group (n = 57) were used to verify the conclusions drawn from the qualitative analysis.

For quantitative analysis, descriptive statistics were used to analyze selection rate and attrition rate using IBM SPSS Statistics 20 (**IBM**, **SPSS** Inc., Chicago, IL).

### Ethical aspects

Eligible dyads were asked to sign an informed consent. Informed consent was asked from both the person with dementia and the caregiver. All participants signed their informed consent (all people with dementia were able to sign their own informed consent). In the Netherlands mental competence is assumed, unless there is reason to doubt or evidence that this is not the case. When judging capacity, the use of cognitive measures such as the MMSE as a proxy for judging capacity is limited [23, 24]. To check the competence of people with dementia in this study, we explained what participation in the study (including homework) would mean for them. In addition, we asked a few open-structured questions in order to verify if they understood and could reproduce the treatment information. They were not included in the study if their answers were not in line with what they had been told and, in these cases, we did not consider them as competent to make their own decision. The study protocol was approved by the Medical Ethics Review Committee of the VU University Medical (registration number 2008/320) and is registered at the Netherlands National Trial Register. Trial number: NTR1802.

## Results

### Study population

#### Recruitment and selection rate

**Figure 1:**
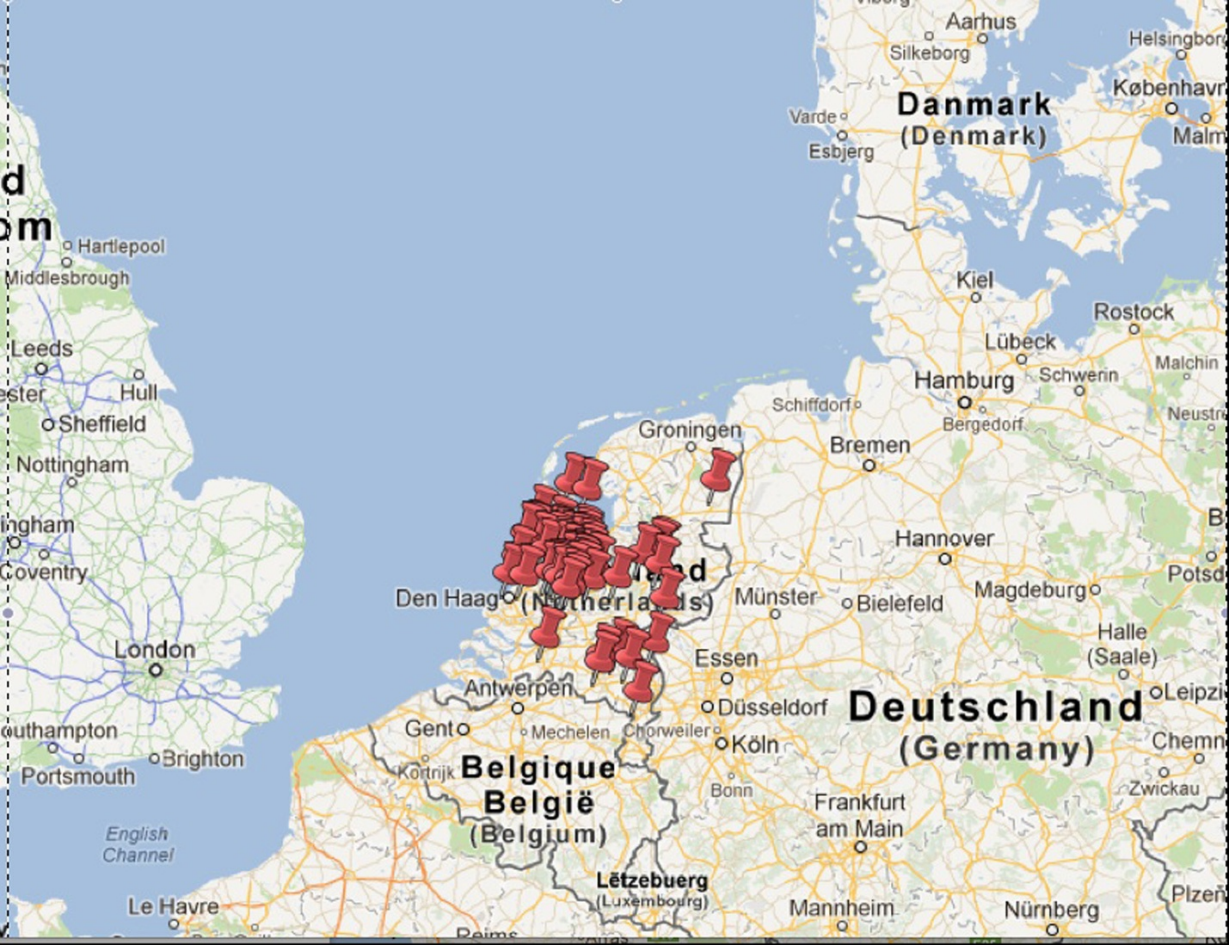
**Recruited eligible dyads throughout the Netherlands.** (Source: Google Maps).

**Figure 2:**
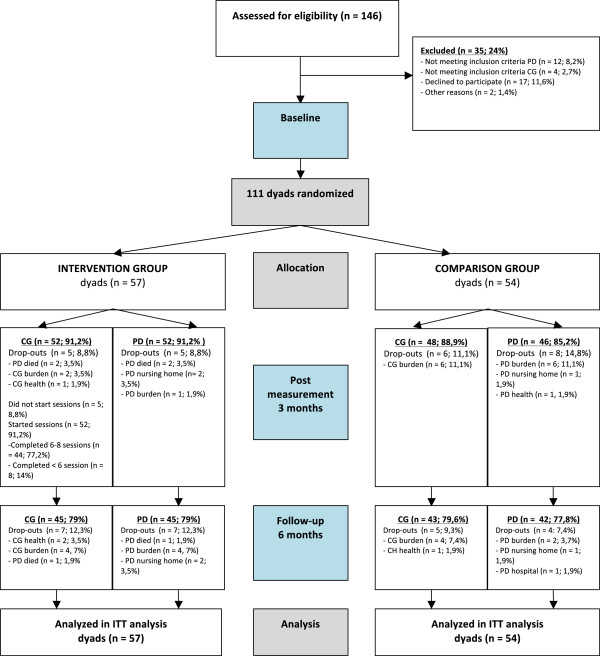
**Flow diagram of the randomized controlled trial.** CG, caregiver; ITT, intention-to-treat; PD, person with dementia.

Recruitment was time consuming and complicated in this geriatric population. We started the recruitment with advertisements in national and local newspapers and on geriatric websites. These advertisements yielded almost no reaction from interested dyads. This was also true for personal letters sent to caregivers of people with dementia via caregiver organizations. Therefore, we changed our recruitment strategy into a personal approach to the dyads by giving presentations at local Alzheimer cafes (public meetings for people with dementia, their caregivers and others). We started with visiting Alzheimer cafes in the neighborhood of Amsterdam. Later, we expanded our visits to Alzheimer cafes throughout the Netherlands. Personally contacting potential participants or case managers was more successful. When contacting case managers, we asked them to look out for potential participants in their own caseload. Case managers contacted clients that seemed to be potential participants for this study and, if they were interested, their permission was asked as to whether the researchers were allowed to contact them. Because of time and financial reasons, we decided to finish the recruitment after 3 years of recruitment efforts (initially we reserved 1 year for the recruitment of all participants). Eventually, we found 146 interested dyads who were screened for eligibility. To detect an effect size of d >0.40 between the experimental and comparison condition with α = 0.05 and β = 0.80, 78 dyads in each group would have been needed, 156 dyads in total (100%). In total, 111 dyads (71% of the number needed) living throughout the Netherlands met all eligibility criteria (Figure 1) and were randomized and included in the intervention or the comparison group (Figure 2).

#### Barriers and facilitators in recruitment and selection process

Barriers for participation of 35 initially interested dyads were diverse. Twelve people with dementia (n = 12) did not meet inclusion criteria because of low MMSE score (<14) and four caregivers (n = 4) did not meet inclusion criteria because of the use of antidepressants. Other reasons for non-participation were based on second thoughts of initially interested dyads about the intensity of the intervention study: the expected participation burden for the caregiver (n = 6) and lack of time of the caregiver (n = 2). Also, non-cooperation by the person with dementia (n = 9), the death of a person with dementia (n = 1) and a negative advice from a neurologist of a person with dementia (this person with dementia personally chose to ask for and follow advice of their physician) to participate in the intervention study (n = 1) were reasons for non-participation. A facilitator in recruitment of dyads was the possibility to exercise during the intervention; this was the most named reason by the dyads to participate in the study. Arguments for participation given by these interested dyads were: the hope of a positive effect of exercise on cognitive functioning, the general idea that exercise is good for body and mind, a need for exercise on doctor’s prescription, the advantages of a ‘home-based’ program and caregivers’ hope for a higher activity level in the person with dementia by doing the exercises. Another facilitator for the recruitment of dyads was participation in scientific research. Dyads indicated that they liked to participate in the intervention in the interest of scientific research, to do something for society.

#### Follow-up: attrition rate and barriers and facilitators for follow-up

In people with dementia, drop-out was mainly due to mortality of the person with dementia, nursing home placement or hospitalization, physical and mental burden and health problems. Drop-out in caregivers was mainly due to perceived burden of providing care, health problems and mortality of their care receiver (Figure 1). Before the first post-measurement, 13 (14%) of people with dementia and 11 (12%) caregivers dropped out (in total 11 dyads: 10%). Before the 6-month follow-up measurement, 13 + 11 (27%) people with dementia had dropped out and 11 + 12 (26%) caregivers had dropped out; in total 23 dyads (21%). Dropout rates for both the intervention group and the comparison group were comparable.

### Multiple intervention components

#### Quality, barriers and facilitators of delivery and adherence of the interventional components

As shown in Table 3, 44 dyads (77.2%) completed all eight home visits. Five dyads (8.8%) did not start with the home visits because of death or nursing home placement of the person with dementia. Reasons for canceling or rescheduling a session were health issues of the person with dementia or caregiver, obligations of the dyads (health care visits, funerals, work obligations of the caregiver), change of days in day-care facility of the person with dementia, and planned holidays of the dyads.

During the home visits, in the presence of the coaches, all dyads were willing to participate in all intervention components, but the dyads differed in performing the intervention components when not in presence of the coaches. In the intervention group, 39 dyads (68.5%) continued to exercise at home and 27 dyads (47.4%) continued to plan pleasant activities after visits by their coach (Table 5). A facilitator to delivery of both the exercise and the support component was the participation of motivated dyads (more specifically in the case of delivery of the exercise component - the participation of sportive dyads and/or healthy dyads). Barriers in delivery of the exercise component were physical complaints and caregiver burden.

**Table 5:** **Compliance to homework (exercise and pleasant activities planning) and presence home visits**

Homework and home visit compliance ***(***n = 57)	n (%)
**Home visit compliance (8 home visits)**	
Completed: 6–8 home visits	44 (77.2)
Partly completed: <6 home visits	8 (14)
Not started with intervention: no home visits	5 (8.8)
**Homework exercise compliance (3 times a week)**	
3 or more times weekly exercise	23 (40.4)
1-2 times weekly exercise	16 (28.1)
0 times weekly exercise (intervention (partly) received)	13 (22.8)
**Planning pleasant activities compliance (without assistance coach over at least 6 weeks)**	
Planned pleasant activities according to protocol: >6 weeks	18 (31.6)
Partly planned pleasant activities: 1–6 weeks	9 (15.8)
No pleasure activities planned (intervention (partly) received)	25 (43.9)
No pleasure activities planned (not started with intervention)	5 (8.8)
**Combined homework and home visit compliance**	
Full compliance according to the protocol: completed 8 home visits, exercised 3 times a week, and planned pleasant activities	9 (15.8)
Moderate compliance	43 (75.4)
Not started with intervention	5 (8.8)

Outside the home visits of the coach, homework (exercises and planning pleasant activities) was not always completed by the dyads as was demonstrated by dyads’ logs. Barriers for homework exercise performance were physical impairments of the person with dementia, time constraints, burden of the caregiver, fatigue of the person with dementia and difficulties in motivating the person with dementia by the caregiver. Barriers for planning pleasant activities were time constraints and burden on the caregiver. These barriers for the performance of homework assignments may indicate too intensive an intervention for this older frail population or a need for more guidance of the dyads by a coach.

#### Experiences of participants with exercise intervention component

Most interviewed participants indicated that they experienced some benefits from the exercises: most people with dementia and almost all caregivers indicated increased pleasure and mood. Some people with dementia and a few caregivers indicated better self-esteem.

“Our coach was surprised I made such good progress in performing the exercises”. (PD 2 proudly said)

Some people with dementia and almost all caregivers mentioned increased awareness of the importance to exercise and some caregivers experienced doing exercises as a pleasant daytime activity in case of bad weather. Furthermore, an interesting mutually indicated benefit of the exercise component was improvement of the quality of the relationship (doing something together increases the confidence in each other) for about a half of the caregivers and some people with dementia.

“Reciprocity and interaction! The good thing of the program was that it was directed to both of us. That was clear from the start of the program. In case of a dementia process, equality between partners should be maintained as long as possible. My partner and I had the idea that we do it together; we can do it together”. (CG 5)

Interviewed participants indicated that different elements incorporated in the intervention components contributed to their experienced benefits. Named elements by both people with dementia and caregivers were the presence of the coach, the use of the ball and the user manual with pictures of the exercises. For some people with dementia and some caregivers (mentioned about their care receiver), the pictures in the user manual worked out as an important mnemonic (sometimes better than verbal instructions) in how to perform the exercises. Almost all caregivers mentioned that remarks of the coach had more influence on the person with dementia than their own remarks in the absence of the coach. Caregivers indicated that the method of the coach was personal, positive, stimulating and with patience for the person with dementia. For some people with dementia and about a half of the caregivers, the exercises with the ball were the most attractive.

“I had not expected that she liked the exercises with the ball the most. She is almost childlike in that; when we go out, the ball has to go with us. This great effect is an eye opener to me. And this soft ball does not damage anything inside the house. I also notice that she becomes happy by playing with the ball. She starts laughing and I start to make jokes”. (CG 9)

Negative working elements were also indicated by interviewed participants. A few caregivers mentioned that doing exercises confronted the person with dementia with physical and mental inabilities. Furthermore, strengthening and balance exercises were indicated to be difficult for people with dementia by some caregivers and people with dementia.

#### Experiences of participants with support intervention component

Thinking about pleasant activities made most caregivers more aware of the importance of these activities.

“After the support of the coach, we do not undertake more activities alone or together. We undertake activities with more awareness”. (CG 2)

“When we now plan an activity, we think more about the experience of the activity for both or one of us”. (CG 1)

Most interviewed caregivers were neutral about the communication skills training. Caregivers mentioned no specific benefits of the communication training, and caregivers mentioned they were already familiar with these communication skills. This was also about the case for psycho-education: about half of the interviewed caregivers mentioned they were already familiar with the educational information provided by the coach as they had already received the same information via case manager visits, at Alzheimer Cafes and via information material from the Dutch Alzheimer Foundation. However, these caregivers also noticed that they experienced this educational repetition as pleasant. Most caregivers indicated that a general benefit of the conversations with the coach was decreased loneliness. This benefit is closely related to received attention, indicated by most caregivers as a working element of the received support.

“Most of the time we see nobody. We just have lonely days. We were looking forward to the visits of our coach”. (CG 7)

Another useful element indicated by a few caregivers was the use of pleasant activities logs.

“Writing down our planned pleasant activities was important to me. Then, afterwards, you only just have to do it”. (CG 9)

An important need mentioned by half of the interviewed caregivers was private time with the coach without the person with dementia being present. During conversations in the presence of the person with dementia, these caregivers indicated they felt controlled by their care receiver when talking about their thoughts and feelings.

“There was time to talk with the coach about problems. However, I couldn’t tell my problems in the presence of my partner, do you understand? That was difficult. I couldn’t speak freely about my thoughts and emotions”. (CG 4)

### Data acquisition

#### Outcome measures: coverage of interventional components

Quantitative outcome measures were directed at mood, burden and general health for caregivers and directed at mood, behavior problems, physical health, cognition and rest-activity rhythm for people with dementia. The qualitative study part revealed that some of the experienced benefits named by the interviewed participants were not specifically assessed as quantitative outcomes (increased pleasure, increased relationship quality, increased self-esteem, decreased loneliness, better awareness of the importance of pleasant activities and exercises and doing exercises as a new daytime activity in case of bad weather).

#### Completeness of data collection

To prevent missing values, examiners were instructed to check the completeness of questionnaires after they had been filled out. There were no missing values among caregivers on the primary outcomes in the baseline measurement. One caregiver had missing values on the primary outcomes in the post-measurement assessment by not filling out the back of the questionnaire. None of the other caregivers had missing values on the primary outcome other than drop-outs in post-measurement or follow-up measurement. In people with dementia, there were no missing values on primary outcomes in all measurements other than drop-outs.

#### Barriers and facilitators for data collection

According to the participants, quantitative measurements were considered as long, but not too burdensome. The measurements for the people with dementia and the questionnaires for caregivers for the quantitative study part were easy to understand and follow.

## Discussion

This process evaluation was conducted to create in-depth insight into the performed exercise and support intervention study designed to reduce depressive symptoms in people with dementia and their family caregivers living in the community. We evaluated the study population, intervention components and data acquisition before the effect analysis based on qualitative and quantitative data according to the framework of Reelick and colleagues [15].

### Study population quality

Evaluation of the study population quality revealed a profound recruitment process, which demonstrated that the modest sample size (though the sample size is reasonable in this field of geriatric research) is due to the difficult accessibility of participants in this frail population and is not due to the tireless efforts of the main researcher. It was difficult to find interested dyads, and therefore the recruitment of participants took longer than originally planned. What did work was recruitment via Alzheimer cafes. However, recruitment efforts may have generated a self-selected group of dyads who were more motivated to exercise. This could have influenced external validity by not approaching dyads who were not familiar with exercise or, for instance, not visiting Alzheimer Cafes. Furthermore, not all interested dyads met inclusion criteria (n = 16) or, for example, dyads were unwilling to participate after receiving more information (n = 8) because of expected burden on the caregiver or lack of time of the caregiver. However, the drop-out rate during follow-up was reasonable (<30%). Conventionally, a 30% drop-out rate for long-term follow-up (≥6 months) studies is regarded as acceptable in this older population dealing with a degenerative dementia process [12]. For people with dementia, the drop-out rate in the study of Teri was similar at post-measurement (caregivers were not measured in the study of Teri and colleagues [14]). Future studies for people with dementia living at home and their family caregivers should account for the barriers to recruiting, such as concerns about time needed from the caregiver. Our sample size was not confined to a particular metropolitan area: dyads throughout the whole country were participating, indicating no geographical bias. Furthermore, there were no complicated informed consent regulations, which may block participation of eligible dyads. In the present study, all eligible dyads, including people with dementia, signed informed consent, which indicated no informed consent bias.

### Multiple intervention components quality

Although most people with dementia and their caregivers experienced benefits of both intervention components (exercise and support) and had high attendance rates to the eight home visits (n = 44), attrition to homework was modest (n = 39 for exercise homework and n = 27 for pleasant activities homework). As shown in Table 5, homework adherence was better for the exercise component than the pleasant event support component of the intervention, which indicates that the exercise component might be better suited to the needs of the participants. With regard to the exercise component, participants experienced pleasure, better mood, more self-esteem, increased awareness of the importance of exercise and improvement in the quality of the relationship. Furthermore, caregivers mentioned that doing exercises was a pleasant daytime activity in case of bad weather. With regard to the support component, named benefits were increased awareness of the importance of pleasant activities and decreased loneliness. There were also disadvantages brought up by participants, which may indicate too intensive an intervention for this frail population: a few caregivers experienced performing the intervention to be a burden because of losing too much of their valuable time and noticed an unpleasant confrontation for physical and mental inabilities in their care receivers by performing (too complicated) exercises. This was due partly to the specific care situation in the Netherlands: most people with dementia living at home receive one or more days respite care in a day-care facility and receive home visits of a case manager (a personal counselor for people with dementia and their caregivers). Together with the visits and homework for the present intervention study, there little free time was left between the caregiver and the person with dementia. For future comparable intervention studies, we advise carefully weighing the burden-benefit ratio in this frail older population and in addition to take note of the fact that usual care can vary by region.

Three quarters of the dyads completed six to eight home visits, which indicates that it was feasible to deliver the intervention at its current frequency and duration. Meanwhile, adherence to homework according to protocol was moderate, being somewhat better for the exercise component than the pleasant activities support component of the intervention. Comparison of these exercise rates with Teri and colleagues [14] showed no large differences. Teri and colleagues do not specifically report the adherence to pleasant event training, although they mentioned that adherence to program recommendations was quite high. This is in contrast to our present evaluation, showing moderate adherence for pleasant activities homework. Future studies should account for the barriers to homework adherence in people with dementia and their caregivers.

Although dyads reported benefits of the intervention, when considering delivery and adherence we conclude that the validity was not the same for all intervention components. Statistical effect analyses should account for this finding: results of the primary and secondary outcome measures should be interpreted with caution.

### Data acquisition

Evaluation of the data acquisition showed a careful collection of data with almost no missing data in primary outcomes and the positive value of the use of a mixed design: qualitative analysis of the intervention revealed outcomes not measured in the quantitative analysis such as pleasure, relationship quality, self-esteem, loneliness and awareness. For future quantitative research in this field, we recommend using instruments with good psychometric properties to measure these ‘experienced’ outcomes, such as quality of life instruments.

### Strengths and weaknesses

The present RCT on the effects of a multicomponent dyadic intervention is a careful and soundly performed study. The type of design used, a mixed-method design, contributed to valuable insights gained from the integration and interpretation of qualitative and quantitative data within one study. Adding qualitative data gives insight into potential underlying working elements as well as diverse perspectives of participants. Furthermore, the framework of Reelick that was used to present the data of the process evaluation contributed to in-depth insight into this complex intervention study, which allows us to draw appropriate conclusions on the process of the intervention and the study.

However, some limitations are also worth considering. A limitation worth noting, because of design considerations, is that we did not involve the experiences of the coaches in the qualitative study. Our main research questions outlined in our protocol [13] were directed at effects and experiences of the participants and not of the coaches. Therefore, we could not compare participants’ experiences with coaches’ experiences. For future effect studies, it is advisable to involve a meticulous process evaluation from the start of the study. Furthermore, because of ethical considerations, we did not collect data of dyads who gave no consent to participate in the intervention study. Therefore we could not compare our study population with non-participating dyads.

## Conclusion

The results on the process evaluation demonstrated a study design and intervention protocol meeting high research standards and giving valuable insights by the integration of qualitative and quantitative data. However, some limitations are also worth considering. Both the exercise and the support component may have different feasibility by moderate attrition to exercise and pleasant activities homework and some negative experiences of participants, which may be an indication of too intensive an intervention for this frail population in this specific region. Results of the statistical effect analysis should be interpreted with caution, accounting for the extent to which homework of both components was performed. In this respect, the importance of a process evaluation before effect analysis and in case of cross-country transmission of a complex intervention is emphasized. In general, the present findings may be useful for those who are designing, implementing, or replicating a similar complex intervention for people with dementia and their caregivers.

## Abbreviations

ABC: Activating events Beliefs Consequences
MMSE: mini-mental state examination
RCT: randomized controlled trial.
